# With an Ear Up against the Wall: An Update on Mechanoperception in *Arabidopsis*

**DOI:** 10.3390/plants10081587

**Published:** 2021-07-31

**Authors:** Sara Behnami, Dario Bonetta

**Affiliations:** Faculty of Science, Ontario Tech University, 2000 Simcoe St N, Oshawa, ON L1G 0C5, Canada; Sara.Behnami@dartmouth.edu

**Keywords:** mechanoperception, cell wall-associated receptor, mechanosensitive channel

## Abstract

Cells interpret mechanical signals and adjust their physiology or development appropriately. In plants, the interface with the outside world is the cell wall, a structure that forms a continuum with the plasma membrane and the cytoskeleton. Mechanical stress from cell wall damage or deformation is interpreted to elicit compensatory responses, hormone signalling, or immune responses. Our understanding of how this is achieved is still evolving; however, we can refer to examples from animals and yeast where more of the details have been worked out. Here, we provide an update on this changing story with a focus on candidate mechanosensitive channels and plasma membrane-localized receptors.

## 1. Introduction

Cells interact with their external environments to sense physical contact, sound waves, gravity, the attachment to other cells and to internal microenvironments which can dictate cell shape [1]. A diversity of molecules mediates the perception of mechanical stimuli. These can include stretch-activated ion channels, mechanosensitive receptors like integrins, growth factor receptors, and G protein-coupled receptors, to name a few [2,3,4]. In this review, we first take a brief look at some examples of how mechanoperception is achieved in prokaryotes, yeast, and animals and then give an update on mechanisms that have been implicated in *Arabidopsis* mechanoperception. An important caveat is that it is often experimentally very challenging to show that a protein is involved in mechanoperception, especially in the right cellular context. Hence, for now, we must be satisfied with candidate molecular players. We limited our review to putative mechanosensitive channels and plasma membrane-localized receptors that might act as cell wall sensors (see Table 1).

How plants perceive physical perturbations has been of interest for many years, and in recent years, there has been much research devoted to this question [5]. Some common elements linking mechanoperception in disparate organisms do exist, but many of the mechanisms that have emerged in plants seem to be unique. These are most likely tied to the nature of plant cell walls and the cellular architecture that distinguishes plants from other eukaryotes. Many of the components that have been implicated in *Arabidopsis* mechanoperception are overlapped with processes associated with immunity, drought, hormone signalling, and development [6,7,8]. While interesting, this feature can often make interpreting the role of individual components more challenging.

**Table 1:** Mechanosensitive channels and cell surface receptors involved in cell wall integrity responses.

Gene	Function	Reference
**Mechanosensitive membrane channels**		
*MSCS-LIKE 1,*	Dissipation of the mitochondrial membrane	[9]
*MSL1* (*At4G00290*)	potential when it becomes too high.	
*MSC-LIKE 2,*	Maintenance of the plastid size and leaf shape.	[10]
*MSL2* (*At5G10490*)		
*MSC-LIKE 3,*	Protection of plastids from hypoosmotic shock.	[11]
*MSL3* (*At1G58200*)		
*MSC-LIKE 8,*	Normal pollen function during hydration and germination.	[12]
*MSL8* (*At2G17010*)		
*MSC-LIKE 10,*	Response to cell swelling.	[13]
*MSL10* (*At5G12080*)		
*MID1-COMPLEMENTING*	Ca^2+^ influx and touch sensing in roots.	[14]
*ACTIVITY 1, MCA1* (*At2G17780*)		
*MID1-COMPLEMENTING*	Normal Ca^2+^ uptake in roots.	[15]
*ACTIVITY 2, MCA2* (*At2G17780*)		
*REDUCED HYPEROSMOLALITY,*	Osmotic Ca^2+^ signalling in guard and root cells.	[16]
*INDUCED CA2+ INCREASE 1,*		
*OSCA1* (*At4G04340*)		
*DEFECTIVE KERNEL 1,*	Required for mechanosensitive Ca^2+^ channel activity.	[17]
*DEK1* (*At1G55350*)		
*PIEZO 1,*	Ca^2+^ influx and touch sensing in roots.	[18]
*PZO1* (*At2G48060*)		
**Cell wall-associated receptor kinases**		
*CELL WALL-ASSOCIATED*	Pectin binding, oligogalacturonide receptor.	[19,20,21]
*KINASE 1, WAK1* (*At1G21250*)	Interacts with glycine-rich protein 3 (GRP3).	
*CELL WALL-ASSOCIATED*	Pectin-activated signalling and gene expression.	[22]
*KINASE 2, WAK2* (*At1G21270*)		
***Catharanthus roseus*** **receptor-like kinases**		
*THESEUS 1,*	Cell expansion, wall integrity maintenance, receptor	[23,24,25]
*THE1* (*At5G54380*)	for RALF34.	
*HERCULES RECEPTOR*	Partially redundant with cell expansion *THE1* activity.	[26]
*KINASE 1, HERK1* (*AT3G46290*)		
*HERCULES RECEPTOR*	Partially redundant with cell expansion *THE1* activity.	[26]
*KINASE 2, HERK2* (*At1G30570*)		
*FERONIA,*	Touch-dependent CA^2+^ signalling, bind pectin.	[27,28]
*FER* (*At3G51550*)		
*ANXUR 1,*	Pollen tube integrity maintenance.	[29]
*ANX1* (*At3G04690*)		
*ANXUR 2,*		
*ANX2* (*At5G28680*)		
**Chimeric leucine-rich repeat/extensin cell wall proteins**		
*LEUCINE-RICH REPEAT/*	Overlapping activities with CrRLK1 proteins, bind RALFs,	[30,31,32]
*EXTENSINS, LRXs*	cell expansion in vegetative tissues, pollen tube integrity.	
**Proline-rich extensin-like receptor kinases**		
*PROLINE-RICH EXTENSIN-*	Root growth, floral organ formation, abscisic acid responses.	[33,34,35]
*LIKE RECEPTOR KINASES,*		
*PERKs*		
**Leucine-rich repeat receptor kinases**		
*FEI 1, FEI1* (*At1G31420*)	Cell wall biosynthesis, ethylene and abscisic acid responses.	[23,36,37,38]
*FEI 2, FEI2* (*At2G35620*)	Cell wall integrity maintenance.	
**Other receptor-like kinases**		
*MDIS1-INTERACTING*	Cell wall integrity, root growth, abiotic and biotic stress	[23,39]
*RECEPTOR LIKE KINASE2,*	responses.	
*MIK2* (*At4G08850*)		
*SCM, SCRAMBLED, SRF9,*	Cell wall integrity responses.	[40]
*STRUBBELIG, STRUBBELIG-*		
*RECEPTOR FAMILY 9,*		
*SUB* (*At1G11130*)		

## 2. Mechanosensitive Channels: From Germs to Worms

Fast responses to mechanical stimuli, which can include external sound, touch, pressure, osmotic pressure (turgor), or membrane bending, have highlighted the importance of mechanosensitive (MS) channels. A classic example of an MS channel was first discovered in giant *E. coli* spheroplasts that were subjected to the patch clamp technique [41]. The pressure exerted by the pipette electrode, which essentially causes stretching of the membrane, activated the channels and induced a current. Pressure could also be elicited by osmotic differences across the membrane [41]. In intact cells, MS channels thus act as safety valves under hypotonic conditions, alleviating turgor pressure extremes by allowing small molecules to leak out of the cell to prevent membrane damage [42]. A genetic link to this phenomenon came from mutations in *E. coli* in two major MS channels, MscL (mechanosensitive channel of large conductance) and MscS (mechanosensitive channel of small conductance) [2,43,44]. Lack of activity of MscL/MscS causes cells to lyse in hypotonic conditions [45]. The MscS channel opens at low-to-intermediate pressures, is homoheptameric, and forms a pore of ~1.3 nm in diameter, with large cytoplasmic domains limiting the molecules reaching the pore [45]. By comparison, the MscL is also pentameric but responds to extreme pressures that might otherwise cause cell rupture and thus has a higher pressure threshold than MscS for its activation [46,47].

In prokaryotes, the channel itself is often sufficient for mechanotransduction. However, identifying equivalent channels in eukaryotes is complicated by their association with protein complexes, the extracellular matrix (ECM) or the cytoskeleton, making them difficult to reconstitute in a test tube or to assay by heterologous expression [2,48,49,50,51]. Nonetheless, examples of what are considered MS channels do exist. These include degenerin and epithelial sodium channels (DEG/ENaC), N-type transient receptor potential (TRPN), two-pore potassium channels (K_2_P), transmembrane-like proteins (TMC), and PIEZO [52,53]. The ability to employ genetic analysis in *Caenorhabditis elegans* made it a natural choice for conducting screenings aimed at identifying mechanically responsive proteins. Indeed, MEC-4 and MEC-10 (mechanosensory abnormality) were identified in screenings for mutants defective in response to gentle touch and are components of one of the earliest examples of a touch-mediated complex in animals [54]. MEC-4 and MEC-10 form a heterodimeric mechanosensitive channel in conjunction with two auxiliary proteins, MEC-2, a stomatin-like protein, and MEC-6, a paraoxonase-like protein [55,56]. The proteins interact electrophysiologically in *Xenopus* oocytes to effect mechanically induced currents [55,57,58]. These studies have been instructive to operationally define what should be considered an MS channel [51]; namely, that the channel is expressed in the right place at the right time, removing the channel removes the MS response, altering the channel properties alters the response, and heterologously expressing the channel shows that it is mechanically gated. While it is difficult to fulfill all these criteria in any system, and there are obvious differences between plants and animals, it does serve as a useful benchmark for moving forward.

A perhaps more relevant example, because of the existence of a cell wall and lack of specialized sensory cells, comes from yeast. In *Saccharomyces cerevisiae*, an important MS mechanism involves a Ca^2+^ influx system that can become activated by hypertonic or hypotonic shock, cold stress, or salt stress [59,60]. It is comprised of two plasma membrane proteins: Cch1 (calcium channel homologue), which is similar to the mammalian α1 pore-forming subunit of L-type voltage-gated calcium channels [61,62], and Mid1 (mating pheromone-induced death), which is an N-glycosylated membrane protein found in the endoplasmic reticulum and plasma membrane, and features homology to α2/δ-like proteins (auxiliary proteins required for voltage-gated calcium channels in animals) [63]. While mutations in either *MID1* or *CCH1* are innocuous when Ca^2+^ is abundant, in low Ca^2+^ conditions, mutated cells die in response to mating pheromones [61,64]. Mid1 confers stretch-activated calcium influx into the yeast cell but also in heterologous expression systems and acts as a sensor for mechanical stress, as well as other stresses [60,61,64,65,66]. Activation of the Cch1–Mid1 complex ultimately leads to the accumulation of intracellular calcium and, in turn, activation of calcineurin, which is a calmodulin-dependent serine/threonine protein phosphatase [67]. Calcineurin targets the Crz1 (calcineurin-responsive zinc finger) transcription factor [68,69], whose transcriptional targets include GSC2/FKS2 (one of the glucan synthases of *S. cerevisiae*) [70,71], providing a mechanism that feeds back onto cell wall remodeling. As we discuss below, proteins with homology to CCH–MID1 do not exist in plants, but functionally equivalent channels have been identified by complementation of yeast mutants [14].

## 3. Mechanoreceptors—Perception at the Extracellular Matrix

The ECM plays a crucial role in how cells interpret their external environment, how they develop, whether they proliferate, what shapes they assume, and how they function. The matrix itself is a complex mixture of polysaccharides and proteins that are secreted locally and elaborated into a network that exists in tight association with the surfaces of the cells that produce it. Depending on its composition, the ECM can also have a supportive function, as with the connective tissues and exoskeletons in animals and the cell walls of plants, fungi, and prokaryotes. While the arrangement of the ECM in animals and the cell walls of plants and fungi are compositionally and structurally different, perception of mechanical stimuli seems to involve cell surface receptors. The mechanisms of converting those mechanical forces in mammalian and yeast examples both involve stretching or compression of the extracellular domains (ECDs) of those receptors [4,72]. In both systems, receptors also become enriched in areas of the cell surface experiencing higher forces [73,74]. Whether similar mechanisms will surface in plants is yet to be established [5].

### 3.1. Mechanoreceptors in Animals

In animal cells, mechanoperception in the ECM is achieved by cell surface receptors that attach cells to the ECM or each other. One of the most important mechanisms to accomplish this hinges on the activity of integrins, which can mediate adhesion to the ECM as well as neighbouring cells [74]. By virtue of their connection to the actin cytoskeleton on their cytoplasmic end, they can communicate mechanical stimuli to the inside of the cell. Cells can adjust internal tension forces and translate mechanical forces into biochemical signals by force-induced conformational changes in mechanosensitive proteins by exposing enzyme-susceptible sites or cause them to unfold (like with integrins), allowing for changes in protein–protein interactions or activity [75].

Integrins can relay external and internal signals in both directions [76]. The structure of integrins is such that an ECD interacts with proteins residing in the ECM and an intracellular domain serves as an attachment site for actin-associated proteins, which form a focal adhesion complex (FAC) [74]. In mammals, they exist as heterodimeric receptors with at least 24 different combinations of interacting α-subunits (18 types) and β-subunits (eight types), which mediate the binding of diverse ECM components [77]. Each subunit is composed of three distinct domains; a large ECD ranging from 700 to 1100 amino acids, a single transmembrane domain (20–30 amino acids) and a small cytoplasmic domain (20–75 amino acids) [78]. The ECDs of both subunits confer ligand binding [79]. The best-understood ligands in the ECM are proteins bearing an RGD or an LDV sequence, which is found in fibronectin and other proteins [79].

Integrin ligand binding, if under the influence of an applied force, affects integrin conformation [78]. Support for this comes from structural studies and simulations, which suggest that integrins exist in inactive and active state conformations [80,81,82]. In the inactive state, the ECDs of the integrin dimer are compact and folded over (bent configuration), making them unable to bind their ligand in the ECM. Cytoplasmic tails in the inactive state are conformationally hooked together and thus prevented from binding to internal ligands. In the active state, the ECDs become unfolded extending the ligand-binding domains located at the ends of the subunits, while the intracellular domains become unhooked to expose binding sites for intracellular ligands [74]. For example, in T cells, application of force to αLβ2 integrins elicits a switch to an active conformation [83].

Another feature of integrins is that they can cluster into adhesion complexes. The components of the FAC include adaptor proteins like talin and paxillin, non-receptor tyrosine kinases (e.g., focal adhesion kinase (FAK)), other tyrosine kinases like Src family kinases, integrin-linked kinases, and vinculin [84]. The association between the ECM and integrins and integrin activation are enhanced by proteins like talin [85]. Force-induced stretching of talin unmasks vinculin-binding sites [86], suggesting that this stretching is a key factor in adhesion complex assembly. Cells are also able to gauge ECM rigidity. Attachment to a rigid ECM capable of resisting strong pulling forces leads to the recruitment of additional integrins and other adhesion complex proteins [87]. In contrast, attachment to a soft ECM produces less tension and a less pronounced response. Therefore, the gauging of ECM rigidity is based on forces that the cell itself imposes on the ECM. While integrin-homologous proteins do not exist in plants, the mechanisms that regulate integrin activity might represent a broader phenomenon. It also points to our relatively limited knowledge of whether monitoring intrinsic tension is also relevant in plants [88,89]. Studies in cell shape determination are particularly relevant in this respect and will be especially informative for understanding how mechanical signals are interpreted [90,91,92,93].

### 3.2. Mechanoreceptors in Yeast

The identification of many components in cell wall integrity monitoring in yeast was performed by virtue of mutant screens that used either chemicals that interfere with cell wall structural components, enzymes that break down the cell wall, or inhibitors of cell wall biosynthesis [94,95,96,97,98,99,100,101,102,103]. These include dyes that bind either chitin (calcofluor white), which is a cross-linking component in the yeast cell wall, or β-1,3-glucan (Congo red), the main structural component of the cell wall, echinocandin inhibitors of glucan synthases (like FKS1/2), enzymes that break down the cell wall (like zymolyase), or caffeine, which impinges on downstream elements of the cell wall integrity pathway. Similarly, mutations that perturb cell wall biosynthesis have also been used to isolate cell wall signalling components [104,105,106]. As we shall discuss, the approach of applying cell wall-disrupting agents has also been used to characterize cell wall integrity signalling in *Arabidopsis* [23].

In *S. cerevisiae*, the group of cell surface proteins that act as mechanosensors have, like integrins, a large ECD, a single transmembrane domain, and a short cytoplasmic domain that interacts with intracellular signalling components. These are typically split into WSC-type sensors that include the products of the *WSC1/SLG1* [107,108], *WSC2*, and *WSC3* genes [107] and MID-type sensors that include the products of the *MID2* and *MTL1* genes [95,109]. The ECD in all the five sensors contains serine/threonine-rich regions which are heavily O-mannosylated, likely causing extension and stiffening of the domain [110]. Atomic force microscopy has indicated that WSC1 behaves like a nanospring in vivo, suggesting that this property is behind its ability to respond to mechanical forces [110]. In addition, WSC1 receptors cluster under stress conditions [73]. Analysis of the WSC1 orthologue in fission yeast (*Schizosaccharomyces pombe*) indicates that WSC1 clustering can occur at sites where force is applied and that clusters dissipate upon removal of the localized force [111]. One of the main differences between WSC- and MID-type sensors can be attributed to the nature of the head group in the ECD, which is cysteine-rich in WSC-type sensors, while in MID-type sensors contains *N*-glycosylated asparagine [112]. This difference is thought to impart different binding affinities for different layers in the cell wall; with the WSC-type contacting the glucan layer and the MID-type contacting the glucan and the mannoprotein layers [112]. Their function as mechanosensors is attributed to their transmembrane domain acting as one anchor and the ECD as the other anchor so that perturbations in the wall or stretching in the membrane can be detected and transduced to downstream signalling components [113].

The cytoplasmic domains of WSC1 and MID2 activate the GDP/GTP exchange factor (GEF), ROM2, which goes on to activate a small GTPase, RHO1 [114]. The signal is transduced by subsequent activation of protein kinase C (PKC1), which is a serine/threonine kinase. Phosphorylation by PKC1 of the mitogen-activated kinase kinase kinase (MAPKKK), BCK1, activates two redundant MAPKKs, MKK1 and MKK2, and finally leads to the phosphorylation of the MAPK, MPK1/SLT2 [114]. Once activated, SLT2 activates transcription of the transcription factors RLM1 and SBF (SWI4/SWI6 complex), which regulate the expression of genes involved in cell wall synthesis and repair [115]. Apart from its involvement in cell wall stress responses, WSC1 is also involved in actin cytoskeleton organization [116], bud emergence [108], regulation of endocytosis [117], and autophagy [118], indicating that it has been co-opted for other responses. While no direct equivalents to WSC-type or MID-type cell wall sensors have been identified in plants, they provide a useful template for the kinds of cell wall sensors that might exist.

## 4. Evidence of Mechanoperception in Plants

Plants have astonishing plasticity when it comes to modifying their overall architecture in response to environmental stimuli. For example, in response to mechanical stimuli like wind, plants become thicker and squatter, presumably making them less prone to damage [119]. Similar outcomes can be achieved in the lab by repeatedly touching plants; the response has been termed thigmomorphogenesis [120,121]. At the molecular level, *TOUCH* genes (*TCH*) were identified more than 30 years ago by screening a cDNA library for genes that were upregulated in response to touch [122]. Later, genome-wide transcriptome profiling in *Arabidopsis* revealed that an excess of 500 genes were turned on within thirty minutes of touch stimulation [123]. Since mechanical stimulation induces a rapid increase in cytoplasmic Ca^2+^, it is perhaps not unexpected that some of the touch-responsive genes encode proteins associated with Ca^2+^ signalling; like calmodulins or other Ca^2+^ binding proteins, which are often associated with stretch-activated channels [3]. Other touch-responsive genes include those that encode proteins related to cell wall synthesis or modification such as xyloglucan endotransglycolylases/hydrolases, arabinogalactan proteins, pectin esterases, cellulose synthases, expansins and extensins [123]. However, a large group of touch-responsive genes are also tightly correlated with disease resistance and even dark responses; highlighting that many of the mechanisms elicited by mechanostimulation share the same downstream effectors as those propagated by other seemingly divergent processes.

As a result of cell wall weakening through mutation or chemical treatments, cell wall perturbations, and possibly mechanical stress, are also associated with corresponding adjustment or compensatory changes in cell wall composition. For example, a reduction in cellulose, which provides much of the mechanical support of the wall, leads to compensatory production of ectopic lignin or pectins [124,125,126,127]. The general idea is that breakup of the cell wall by enzyme treatment or the depletion of cellulose by chemical inhibition or mutations in cellulose biosynthesis genes leads to the mechanical breakdown of the cell wall and/or its connection to the plasma membrane. This elicits responses such as the production of jasmonic acid (JA), abscisic acid (ABA), salicylic acid (SA), ectopic lignin and changes the expression of mechanoresponsive genes, immunity-related genes, and cell wall synthesis-related genes [128,129,130,131,132,133,134]. Since this syndrome can be suppressed by the addition of osmotic agents, it is likely the result of mechanical stimulation caused by turgor pressure. It is not clear at this point whether responses to a weakened cell wall are primarily mediated by mechanosensitive channels responding to plasma membrane stretching as a result of the increased pliability of the wall or whether it is via cell wall sensors or, more than likely, a combination of both.

## 5. Mechanosensitive Channels in *Arabidopsis*

In *Arabidopsis*, a number of mechanosensitive channels which are functionally similar to either bacterial MscS or yeast Cch1/Mid1 have been identified. Indeed, MscS-like channels (MSLs) have wide distribution in different phyla including plants [135]. In *Arabidopsis*, there are ten *MSL* gene family members, *MSL1–10*, which vary in sequence identities ranging from 21% to 66%. The structure of MSL1 has recently been resolved using cryogenic electron microscopy indicating that it has an overall structure similar to MscS; with some differences in the transmembrane domain and the matrix domain [136]. Based on their residency in different subcellular compartments as well as tissue-specific expression, it is expected that MSL proteins likely have varied physiological functions. For example, MSL1 is found in mitochondria, MSL2 and MSL3—in chloroplasts, while others—in the plasma membrane or the endoplasmic reticulum [137]. In chloroplasts, MSL2 and MSL3 have overlapping functions in the maintenance of the plastid shape, size, and division [11,135,138]. In pollen, MSL8 is required for pollen grain survival upon rehydration as well as tube integrity during germination and elongation [12,139]. Indeed, eliminating MSL8 function leads to higher rates of bursting of pollen upon hydration and germination, while its overexpression inhibits pollen germination and prevents it from bursting [139]. Plants carrying loss-of-function (LOF) alleles in plasma membrane-localized MSLs have decidedly more subtle phenotypes. While MS channel activity is compromised, more pronounced phenotypes are not obvious even in quintuple mutants [140]. Some members of this class of MSL proteins influence overall plant growth, pathogen responses, and reactive oxygen species accumulation [141,142,143,144]. Determining the precise role of all these diverse proteins awaits further investigation.

Functional complementation of the yeast Mid1 protein led to the identification of an *Arabidopsis* counterpart, which, despite bearing little sequence or structural homology to Mid1, can act on its own to complement not just the *mid1* mutant, but also the *cch1;mid1* double mutant [14]. The plant protein, named MID1-COMPLEMENTING ACTIVITY 1 (MCA1), is plasma membrane-localized like its yeast counterpart and, as well as promoting calcium influx, mediates responses to mechanical and osmotic stimuli [14]. The second protein, MCA2, shares greater than 70% amino acid sequence identity with MCA1 and has similar structural features [145]. Both proteins are single-pass type I integral membrane proteins with a small N-terminal ECD and a larger intracellular region harbouring an EF hand-like region, a central coiled-coiled motif, and a C-terminal PLAC8 (placenta-specific gene 8) motif [146]. Heterologous expression of MCA2 coupled with single particle analysis of cryogenic electron microscopy confirms this conformation and indicates that the protein forms a homotetramer [147]. Although it is expected that the EF hand is regulated by cytoplasmic calcium, the function of the coiled-coil or PLAC8 motifs await description. When MCA1 is expressed in frog oocytes, it imparts increased MS channel activity, which can be triggered by either hypoosmotic shock or by membrane stretch caused by pipette suction [148]. Loss-of-function *mca1* and *mca2* alleles change touch-related root behaviours, indicating that the proteins are required for mechanosensation in vivo [14]. In addition, LOF mutations in the two genes also lead to cold sensitivity, lower cold-induced cytosolic calcium accumulation, and downregulation of cold-induced genes, suggesting that the two MCAs are also required for cold tolerance responses [149]. Recent studies have also implicated MCA1 and MCA2 in the perception of gravity and their role in relaying a biphasic increase in cytosolic calcium in response to gravity [150,151,152].

Apart from MSLs and MCAs, calcium gating is also achieved by the mechanosensitive channel proteins, REDUCED HYPEROSMOLARITY, INDUCED CALCIUM INCREASE (OSCA) [16]. In *Arabidopsis*, there are 15 *OSCA* genes, and this class of channels is conserved among eukaryotes [153]. Mutations in the *OSCA1* gene cause impaired calcium signalling in guard cells and root cells as well as reduced transpiration and root growth under osmotic stress [16]. OSCA1 proteins form dimers and are structurally similar to mammalian transmembrane proteins 16, TMEM16, which are also calcium-activated chloride channels [154]. Although OSCA genes were originally identified based on hyperosmolarity responses, when assayed using electrophysiology in mechanically-insensitive animal cell lines, five out of the six OSCA proteins tested were shown to be mechanosensitive [153]. While many OSCA proteins are important for mechanoperception, *OSCA3* (*EARLY-RESPONSIVE TO DEHYDRATION 4*, *ERD4*) was identified in screening aimed at isolating cDNAs that are upregulated in response to dehydration, suggesting a range of functions for the proteins [155].

Recently, it has been recognized that the DEFECTIVE KERNERL1 (DEK1) protein, which is required for embryogenesis and other developmental processes [156], is also associated with rapid calcium-activated MS channel activity (RMA) in *Arabidopsis* protoplasts [17]. Since *DEK1* has not been expressed in a heterologous system, it has not been confirmed whether it encodes an MS channel or a regulator of a channel conferring RMA activity. The single *Arabidopsis DEK1* gene is predicted to encode a protein with 21 transmembrane helices (TMs), a cytosolic linker region, and a carboxy-terminal calpain domain, which is found in calcium-dependent cysteine proteases [157]. Indeed, the membrane-localized DEK1 protein undergoes autolytic cleavage and the calpain domain is released into the cytosol [158]. While DEK1 activity is associated with epidermal cell differentiation [159,160], embryo development [161], and cell division patterns [162], DEK1 activity also affects cell wall composition by primarily affecting cellulose and pectin deposition [163]. Surprisingly, the calpain domain itself is sufficient to complement *dek1* mutants [158]. In mammals, calpains participate in a diversity of pathways required for cell motility and integrin receptor-linked cell adhesion, to name just two [164,165]. It is worth noting that calpains are downstream effectors of mechanosensitive channels like PIEZO [166], raising the question of whether DEK1 has a combined PIEZO-like/calpain function.

Mechanical resistance can also be caused by heterogeneous soil components which can alter root extension and penetration into soil [167]. For example, a recent study has shown that the *Arabidopsis* PIEZO protein (PZO1) is required for root penetration into compacted soil [18]. The PZO1 protein improves mechanical pressure in columella cells by transducing Ca^2+^ signals to the root elongation zone and/or by protecting cell wall integrity [18]. These results suggest that PZO1 has a mechanosensory function and that mechanotransduction is involved in root responses to mechanical stresses imposed on them by the substrate in which they grow. Additional studies will likely elaborate how the PZO1 protein is mechanistically controlled.

## 6. Candidate Cell Wall Receptors in *Arabidopsis*

Compared to mechanosensitive channels, the identification of cell surface receptors that are linked to mechanoperception in *Arabidopsis* has been more elusive. Although connections between the cell wall and the plasma membrane as evidenced by the formation of Hechtian strands [168] (cytoplasmic threads formed during plasmolysis) have been known for a long time, the nature of these connections remain unclear [169,170]. Interestingly, a LOF mutation in the *NON-RACE-SPECIFIC DISEASE RESISTANCE1* (*NDR1*) gene, which has some homology to mammalian integrins, leads to the loss of plasma membrane–cell wall adhesion [171]. Whether NDR1 fulfills a similar function to integrins is, however, still speculative.

In the following section, we focused on cell surface proteins that have been implicated in aspects of cell wall integrity signalling and in this sense are cell wall sensors. However, it has not been established whether any of these are mechanically sensitive in the same way as the WSC/MID-type or integrins are in yeast and mammals. For example, both WSC/MID-type sensors and integrins are linked to the wall or the ECM and respond to wall or ECM perturbations because their ECDs change conformationally [75,116]. In addition to triggering intracellular signalling, another shared feature is that the receptors are linked to the cytoskeleton. In yeast, WSC1 clustering is mediated by the actin cytoskeleton, while cell wall damage-induced actin depolymerization is mediated by WSC1 [172]. In a similar way, in mammalian cells, integrin clustering is mediated by the actin cytoskeleton, while integrin-mediated adhesion is influenced by actin dynamics [173]. In the case of *Arabidopsis*, we do not yet know if any of the receptors that are discussed below share these features. Nevertheless, the receptors are cell wall-associated, are structurally unique, and possess activities that make them good candidates to have equivalent functions to the yeast and mammalian counterparts. Determining if any of them can indeed respond to mechanical stimuli is technically challenging, especially in the context of the cell wall; however, continued efforts and new techniques will undoubtedly yield more insights into how they operate mechanistically.

### 6.1. Wall-Associated Receptor Kinases

The existence of wall-associated receptor kinases (WAKs) has been known for at least 25 years [174]. Their tight connection with the cell wall based on immunocytochemistry and their fractionation with insoluble materials makes them unique among receptor kinases [174]. Structurally, they possess an ECD, which contains epidermal growth factor (EGF)-like repeats and which is embedded in the wall, a TM region tethering them to the PM, and a cytoplasmic domain kinase domain; making them well-suited to be wall sensors [175]. In *Arabidopsis*, there are five highly similar genes, *WAK1–5*, which differ in their tissue expression and in the structure of their ECDs, suggesting that they have varied roles in plant development and physiology [176]. WAKs are among the few plant receptors for which a cell wall binding ligand has been experimentally discovered. For example, a peptide corresponding to the N-terminal portion of the ECD of WAK1 can bind purified pectin via charged amino acid residues and charged oxygen groups in the pectin [19,177]. While the WAK1 ECD binds to homogalacturonan (HG), oligogalacturonides (OGs), and related alginates, it preferentially does so under cross-linking conditions brought about by the addition of calcium to the pectins [177]. The ECD of WAK2 behaves in a similar way but with less affinity than WAK1 for pectin, and neither has much binding affinity for branched pectins like rhamnogalacturonan I or rhamnogalacturonan II [22].

The ability of WAK1 to bind OGs in vitro and its upregulation in response to OGs [178] prompted Brutus et al. [20] to test whether it behaves as an OG receptor in vivo. Released OGs from the cell wall are damage-associated molecular patterns (DAMPs) which activate plant immune responses [178,179]. Since reverse genetic approaches, either because of redundancy or lethal effects [180,181], limited the spectrum of available *wak* phenotypes, the authors opted to create chimeric receptors to assess whether WAK1 perceives OGs as signal ligands. The approach involved combining the WAK1 ECD with the intracellular kinase domain of the EF-Tu receptor (EFR), a leucine-rich repeat receptor kinase for microbe-associated molecular pattern recognition [20]. The chimeric receptor was capable of sensing OGs in vivo and the WAK ECD could activate the EFR kinase domain, thereby eliciting defence responses that are normally brought about by OGs. Regulation of the response to OGs also involves WAK1 binding a glycine-rich extracellular protein, GRP3, and association of WAK1–GRP3 with a kinase-associated protein phosphatase, KAPP [182]. While WAK1 functions to transduce the OG-triggered signalling, eliciting the expression of defense genes, GRP3 and KAPP act to depress the signal to downregulate immune responses [21]. Whether WAKs are multifunctional, relaying information related to pathogen responses as well as cell wall perturbations, is not known. In addition, whether post-translational modifications [183] play a part in WAK protein functionality also needs to be determined.

### 6.2. THESEUS (and Other Mythical Characters)

An important member of the cell wall sensor family is the receptor kinase THESEUS1 (THE1) [24]. THE1 is one of the 17 receptor kinases belonging to the *Catharanthus roseus* receptor-like kinase 1 (CrRLK1) subfamily of plasma membrane-bound receptor-like kinases, which have ECDs that possess regions that are similar to animal malectin domains [184]. The *THE1* gene was identified as a suppressor of a cellulose synthase 6 (CESA6) mutation (*cesa6^prc1-1^*); an allele which causes plants to have shortened hypocotyls in the dark, accumulate ectopic lignin and callose, and become hypersensitive to cellulose biosynthesis inhibitors [124,185,186]. While the shortened hypocotyls and ectopic lignification in *cesa6^prc1-1^* plants are partly suppressed by LOF *the1* alleles, cellulose deficiency is not, implying that cellulose production is not an obligatory requirement for cell expansion [24]. Conversely, overexpression of a functional THE1–green fluorescent protein (GFP) fusion exacerbates growth phenotypes and ectopic lignification in LOF *cesa6* and *cesa3* mutant backgrounds [24]. More recently, a genetic study using loss- and gain-of-function alleles of *THE1* has confirmed that *THE1* function is required for suppressing cell expansion in response to cell wall damage; caused by either mutation or chemical inhibition [187]. Two other related CrRLK1s, HERCULES1 and 2 (HERK1 and HERK2), are likely to be partly redundant with THE1, since combining the LOF mutations *herk1* or *herk2-1* with LOF alleles of *the1* leads to additive effects with respect to cell expansion, suggesting that in this respect, they work in parallel pathways [26].

*THE1* function is also more broadly required for pathogen responses as well as cell wall damage-related responses. For example, THE1 interacts with a guanine exchange factor (GEF4), operating in the same pathway regulating defense responses to the fungus *Botrytis cinerea* [188]. It is also required for responses to cell wall damage induced by cellulose biosynthesis inhibitors like isoxaben or cell wall-degrading enzymes like Driselase, which both lead to the production of ectopic lignin, jasmonic acid, or salicylic acid [23,188]. Importantly, *THE1* is thought to act genetically upstream of *MCA1* in response to isoxaben-induced cell wall damage, suggesting that perception by THE1 and MCA1 is cooperative [23]. Furthermore, a known ligand for THE1, rapid alkalinization factor 34 (RAFL34), is a peptide which belongs to the family of signalling peptides that have been implicated in a diversity of physiological responses in plants [25,189,190]. In *Arabidopsis*, *RALF* genes include an excess of 30 members [191]. The RALFs that have been tested in cell culture (including RALF34) show strong alkalizing activity (the exception is the pollen-specific RALF4) [25,192]. Indeed, alkaline pH is required for RALF34 binding to the ECD of THE1 as well as its alkalizing activity in the cell wall [25,192]. With respect to monitoring cell wall perturbations, whether changes in the apoplastic context cause the affinity for RALFs to switch from cell wall matrix components to cell surface receptors like THE1 remains to be determined [189,193].

Another member of the CrRLK1 family, FERONIA (FER), has been implicated in an even greater diversity of processes; like tip growth and cell wall integrity in pollen tubes [194,195] and root hairs [196,197], pathogen responses [198,199,200], salt tolerance [27,201], and cell expansion [30,193,202], to name just a few (see [6,203] for review). In the context of mechanical stimulation, LOF *fer* alleles exhibit impaired calcium signalling in response to touch or bending stimulation as well as a decrease in the expression of touch-responsive genes [28]. In addition, mutant plants have root skewing and growth phenotypes such as the inability to penetrate hard agar or bending around obstacles, consistent with a dysfunction in mechanical responses. The ECD of FER, like that of WAK1, has been shown to bind pectin in vitro, which would provide a direct link to the cell wall if the same interaction is true in vivo [27]. Like THE1, FER is also a RALF receptor, with the specificity RALF-binding being dependent on the physiological context [193,204,205]. For example, binding of RALF23 to a complex consisting of FER and a glycosylphosphatidylinositol-anchored protein (GPI-AP), LORELEI-like GPI-AP1 (LLG1), inhibits the EF-Tu receptor (EFR)/FLAGELLIN SENSING2 (FLG2)/BRI1-associated kinase1 (BAK1) complex, which is activated by the flagellin epitope (flg22) to elicit immune responses [204,206]. Binding of FER to RALF1 is instead associated with growth, stress and salinity responses, and stomatal signalling [207,208,209,210,211]. The pleiotropic effects of FER mutations indicate that it is a multifunctional protein, which seems to be a hallmark of cell wall-interacting receptors.

Still other members of the CrRLK1 family like ANXUR receptor kinases (ANX1 and ANX2) have been implicated in cell wall integrity maintenance. Like other CrRLK1 family receptors, these are associated with responses related to resistance to pathogens as well as pollen tube integrity [29,212]. In the context of pollen tube wall integrity, ANX receptors are thought to act upstream of membrane-bound NADPH oxidases, which produce reactive oxygen species (ROS) [29]. Disruption of ANX or NADPH oxidase function leads to NADPH oxidase-dependent ROS production, Ca^2+^ channel opening, and irregular exocytosis of the wall material. The outcome of this disruption, if not stabilized by compensatory mechanisms, is a reduction in cell wall thickness, which decreases to a point where turgor pressure leads to pollen tube rupture [29]. In this context, ANXUR receptors thus act as cell wall sensors required for cell wall integrity maintenance.

### 6.3. Leucine-Rich Repeat Extensin Proteins

Another important connection of FER to the cell wall might be through its interaction with leucine-rich repeat extensin (LRX) proteins [31]. These proteins have an N-terminal signal peptide followed by a leucine-rich repeat (LRR) domain, a cysteine-rich domain, and a carboxy-terminal extensin domain [31,213]. Extensins are a group of proteins with a signature Ser-Hyp_n_ motif that have been associated with increasing the tensile strength of the wall. They are differentially post-translationally modified so that their physical properties affect how they are cross-linked to other cell wall matrix components [214]. Classic extensins become insolubilized in the cell wall matrix through the action of peroxidases and the establishment of intra- and intermolecular crosslinks, involving mainly tyrosine residues [215]. In contrast, the extensin motif in LRXs is variable, and tyrosine residues are required for LRX function but not for LRX insolubilization in the cell wall [216]. Although the exact nature of the extensin domain in LRXs is not fully understood, its existence implies that it serves to anchor the protein to the cell wall. *Arabidopsis* encodes eleven *LRX* genes (*LRX1–LRX11*) which are differentially expressed in either vegetative tissues (*LRX1–LRX7*) or in pollen (*LRX8–LRX11*) [213]. The LRR domain of LRX4 has been shown to interact with the ECD of FER, thus providing a connection between the plasma membrane and the cell wall through the extensin domain [30]. Based on yeast two-hybrid or immunoprecipitation experiments, the LRR domains of other vegetative LRXs (LRX2, LRX3, and LRX5) also interact with FER [32]. These interactions are supported genetically by the similarity in phenotypes of LOF *fer-4* and an *lrx12345* quintuple mutant [32].

Monitoring of cell wall status by FER and LRXs has been demonstrated by inhibiting pectin methyl esterases (PMEs), which are thought to cause cell wall rigidification [30,217]. For example, the application of epigallocatechin gallate (EGCG), an inhibitor of PMEs, interferes with vacuole expansion in growing cells, and this effect is diminished in *fer* or *lrx* mutants [30]. While the membrane association of LRX4 depends on the LRR domain, it does not necessarily require to be associated with FER since LRX4 lacking the extensin domain is still localized to the plasma membrane even in an *fer-4* background [32]. Given that it is unlikely that the LRR domain is sufficient for membrane localization, this aspect of LRX4 suggests that its membrane association might involve proteins other than FER. In addition, the LRR domain mediates the dimerization of LRXs and is functionally required both in vitro and in vivo [218]. Another layer of regulatory complexity is the ability of LRXs to differentially bind RALFs, an aspect of their regulation which is also true for THE1 and FER [30,201]. For example, RALF1 can bind LRX1, LRX4, and LRX5; the same RALF that is also differentially bound by FER [32]. At least for LRX1–FER, RALF1 does not interfere with FER interaction with LRX1 [32]. The relationships between these various components imply that a certain level of combinatorial control is at play, which requires additional experimentation to fully work out.

A far less well-characterized class of related proteins are PROLINE-RICH EXTENSIN-LIKE RECEPTOR KINASES or PERKs. By virtue of their ECDs, which contain extensin-like and arabinogalactan-like motifs, this small group of receptors might also act as cell wall sensors [219,220]. Mutational analysis of a number of PERKs indicates that they are involved in floral organ development [33], root growth [34], and root hair growth [221]. Possible PERK downstream signalling components include KCBP-INTERACTING PROTEIN KINASE1 (KIPK1) and KIPK2 and kinesin-line calmodulin-binding protein (KCBP) [34], which play a role in cell shape determination [222]. Although PERKs have so far not been shown to act as cell wall sensors, their structure warrants that they be given a closer look.

### 6.4. Leucine-Rich Repeat Receptor Kinases

Another important class of receptors related to cell wall signalling are the leucine-rich repeat RLKs, FEI1 and FEI2 [36,223,224]. Indeed, double *fei1;fei2* mutants are impaired in cellulose biosynthesis, which is phenotypically observable as root swelling and is enhanced by supplementing growth media with sucrose or isoxaben [36]. Similar conditional phenotypes are observed in other mutants affecting cellulose biosynthesis like *cesa6^prc1^* [124], *cesa1/rsw1* [225], *cobra* (*cob*) [226], and *salt-overly sensitive5* (*sos5*)/*fasciclin-like arabinogalactan protein4* (*fla4*) [37]. While *fei1;fei2* triple-mutant combinations with *cesa6^prc1^*, *rsw1*, *cob* are additive, suggesting that they work in parallel pathways, the *fei1;fei2;fla4* triple mutant is nonadditive, suggesting that FEI1/2 and FLA4 work in the same pathway [37]. The involvement of FLA4–FEI in cellulose biosynthesis is not limited to roots but also to cellulosic rays in the seed coat of *Arabidopsis* [227], indicating that the complex has a broad effect on cellulose biosynthesis. Interestingly, the *fei1;fei2* phenotypes are suppressed by inhibition of the ethylene precursor 1-aminocyclopropane-1-carboxylic acid or by mutations affecting auxin biosynthesis, which incidentally also supress cellulose deficiency phenotypes in *cesa6^prc1^*, *cob*, and *sos5/fla4* [36,228], linking cellulose synthesis with ethylene and auxin signalling. In the case of FLA4, excess abscisic acid (ABA) or increased ABA signalling suppresses the root phenotypes of *fla4*, while inhibition of ABA synthesis phenocopies the salt-sensitivity phenotype of *fla4*, thereby also linking FLA4–FEI signalling with ABA-related responses [38].

The link between the FLA4–FEI pathway and its possible role in cell wall integrity has been recognized by phenotypic analysis based on responses to isoxaben and/or Driselase [23]. The output from either treatment is similar: accumulation of JA, SA, and lignin. On the basis of these outputs, *fei2* plants are impaired in their response to isoxaben treatment, with lower accumulation of JA, SA, and lignin compared to the wild type, indicating that *FEI2* is involved in cell wall damage perception [23]. Similar reductions in responses to isoxaben are also observed in LOF *the1-1* (as noted earlier) and *mca1* plants. When these mutations are combined to generate *mca1;fei2*, *the1-1;mca1*, or *the1-1;fei2* double-mutant plants, none of them have additive phenotypes. The *mca1;fei2* plants are identical to *fei2* with respect to JA, SA, and lignin accumulation, while the *the1-1;mca1* and *the1-1;fei2* combinations accumulate levels of JA, SA, and lignin similar to the *the1-1* plants [23]. To establish epistatic relationships, *mca1* and *fei2* alleles were combined with the gain-of-function *the1-4* allele [187], which causes plants to accumulate JA, SA, and lignin to a greater extent than the wild type. In both cases, JA and SA levels in the double mutants (*mac1;the1-4* or *fei2;the1-4*) were comparable to the wild-type levels, which suggests that *THE1* might act upstream of both *MCA1* and *FEI2*. It is not clear, however, why double-mutant combinations with *the1-4* do not have JA/SA levels that are more similar to those of single *mca1* or *fei2* plants. The *the1-4* allele is a T-DNA mutant harbouring a predicted truncated version of the THE1 protein, which is missing the intracellular kinase domain but which retains the extracellular and transmembrane domains. These modifications lead to enhanced responses comparable to full-length THE1 overexpression lines [187]. Whether the THE1 ECD is promiscuous and interacts with other receptor ECDs remains to be determined. Regardless, these findings open many interesting possibilities about how THE1 is regulated and how it participates in cell wall sensing.

Another mutant allele tested by Engelsdorf et al. [23] that showed an attenuated response to isoxaben treatment was *mik2-1*. The *MIK2/LRR-KISS* (*LRR-RK MALE DISCOVERER 1-INTERACTING RECEPTOR-LIKE KINASE 2/LEUCINE-RICH REPEAT KINASE FAMILY PROTEIN INDUCED BY SALT STRESS*), or, more simply MIK2, had already been known to influence cell wall damage response signalling [39]. A double-mutant combination of *mik2-1;the1-1* LOF alleles causes a reduction in JA/SA in response to isoxaben treatment similar to *the1-1*, suggesting that the two genes might function in the same pathway. In addition, *mik2-1* plants exhibit changes in root skewing which are dependent on functional THE1 and cellulose synthase activity [39]. MIK2 activity is also required for salt stress tolerance, and this, again, requires functional THE1 [39]. These observations suggest that *THE1* and *MIK2* have distinct, but also intersecting functions. The exact nature of the mechanisms underlying these functions awaits further elucidation.

Another receptor that plays a part in isoxaben-induced cell wall damage responses is LRR-RK STRUBBELIG (SUB)/SCRAMBLED (SCM) [40]. It was previously shown that SUB is required for developmental programs like floral morphogenesis [229], leaf development [230], root hair patterning [231] (see [232] for review). Like *THE1* and *MIK2*, *SUB* function is also required for lignin accumulation in response to isoxaben treatment [40]. However, unlike *MIK2* and *THE1*, *SUB* function is not required for isoxaben-induced JA accumulation. Interestingly, SUB activity is required for cell size and shape maintenance and recovery of root growth following isoxaben exposure, a feature that is not attributed to THE1 function. Based on these and other observations, *SUB* plays a role in cell wall damage signalling in parallel with THE1 and MIK2 to activate some common downstream compensatory responses [40].

## 7. Conclusions

Different mechanisms have evolved to perceive mechanical stimuli in different phyla. The anatomy of plant cells compared to animal and fungal cells certainly plays a part in these differences. Plant cells also experience much higher levels of turgor pressure compared to animal cells, which influences how mechanical stimuli are perceived [233]. Cell walls of plants are also attached to each other, forming a cell wall continuum, which influences perception in whole tissues. Whether perception of external mechanical signals is predominantly the domain of mechanosensitive channels and cell wall-associated receptors act downstream remains to be seen. However, for now, there seems to be a considerable overlap between the two as well as an overlap between the functions of different putative cell wall sensors. Cell wall-embedded proteins, which we largely ignored in this review, are also important in how plant cells are able to perceive cell wall perturbations. A growing number of candidate mechanosensitive channels and receptors exist, and these will need to be further tested to determine exactly how mechanical signals are interpreted.

## References

[1] Vogel V., Sheetz M. (2006). Local force and geometry sensing regulate cell functions. Nat. Rev. Mol. Cell Biol..

[2] Kung C., Martinac B., Sukharev S. (2010). Mechanosensitive Channels in Microbes. Annu. Rev. Microbiol..

[3] Árnadóttir J., Chalfie M. (2010). Eukaryotic Mechanosensitive Channels. Annu. Rev. Biophys..

[4] Luis Alonso J., Goldmann W.H. (2016). Cellular mechanotransduction. AIMS Biophys..

[5] Bacete L., Hamann T. (2020). The role of mechanoperception in plant cell wall integrity maintenance. Plants.

[6] Wolf S. (2017). Plant cell wall signalling and receptor-like kinases. Biochem. J..

[7] Basu D., Haswell E.S. (2017). Plant mechanosensitive ion channels: An ocean of possibilities. Curr. Opin. Plant Biol..

[8] Vaahtera L., Schulz J., Hamann T. (2019). Cell wall integrity maintenance during plant development and interaction with the environment. Nat. Plants.

[9] Lee C.P., Maksaev G., Jensen G.S., Murcha M.W., Wilson M.E., Fricker M., Hell R., Haswell E.S., Millar A.H., Sweetlove L.J. (2016). MSL1 is a mechanosensitive ion channel that dissipates mitochondrial membrane potential and maintains redox homeostasis in mitochondria during abiotic stress. Plant J..

[10] Jensen G.S., Haswell E.S. (2012). Functional analysis of conserved motifs in the mechanosensitive channel homolog MscS-Like2 from Arabidopsis thaliana. PLoS ONE.

[11] Veley K.M., Marshburn S., Clure C.E., Haswell E.S. (2012). Mechanosensitive channels protect plastids from hypoosmotic stress during normal plant growth. Curr. Biol..

[12] Hamilton E.S., Haswell E.S. (2017). The Tension-sensitive Ion Transport Activity of MSL8 is Critical for its Function in Pollen Hydration and Germination. Plant Cell Physiol..

[13] Basu D., Haswell E.S. (2020). The Mechanosensitive Ion Channel MSL10 Potentiates Responses to Cell Swelling in Arabidopsis Seedlings. Curr. Biol..

[14] Nakagawa Y., Katagiri T., Shinozaki K., Qi Z., Tatsumi H., Furuichi T., Kishigami A., Sokabe M., Kojima I., Sato S. (2007). Arabidopsis plasma membrane protein crucial for Ca^2+^ influx and touch sensing in roots. Proc. Natl. Acad. Sci. USA.

[15] Yamanaka T., Nakagawa Y., Mori K., Nakano M., Imamura T., Kataoka H., Terashima A., Iida K., Kojima I., Katagiri T. (2010). MCA1 and MCA2 that mediate Ca^2+^ uptake have distinct and overlapping roles in Arabidopsis. Plant Physiol..

[16] Yuan F., Yang H., Xue Y., Kong D., Ye R., Li C., Zhang J., Theprungsirikul L., Shrift T., Krichilsky B. (2014). OSCA1 mediates osmotic-stress-evoked Ca^2+^ increases vital for osmosensing in Arabidopsis. Nature.

[17] Tran D., Galletti R., Neumann E.D., Dubois A., Sharif-Naeini R., Geitmann A., Frachisse J.-M., Hamant O., Ingram G.C. (2017). A mechanosensitive Ca^2+^ channel activity is dependent on the developmental regulator DEK1. Nat. Commun..

[18] Mousavi S.A.R., Dubin A.E., Zeng W.-Z., Coombs A.M., Do K., Ghadiri D.A., Keenan W.T., Ge C., Zhao Y., Patapoutian A. (2021). PIEZO ion channel is required for root mechanotransduction in Arabidopsis thaliana. Proc. Natl. Acad. Sci. USA.

[19] Decreux A., Thomas A., Spies B., Brasseur R., van Cutsem P., Messiaen J. (2006). In vitro characterization of the homogalacturonan-binding domain of the wall-associated kinase WAK1 using site-directed mutagenesis. Phytochemistry.

[20] Brutus A., Sicilia F., Macone A., Cervone F., Lorenzo G. (2010). De A domain swap approach reveals a role of the plant wall-associated kinase 1 (WAK1) as a receptor of oligogalacturonides. Proc. Natl. Acad. Sci. USA.

[21] Gramegna G., Modesti V., Savatin D.V., Sicilia F., Cervone F., Lorenzo G. (2016). De GRP-3 and KAPP, encoding interactors of WAK1, negatively affect defense responses induced by oligogalacturonides and local response to wounding. J. Exp. Bot..

[22] Kohorn B.D., Johansen S., Shishido A., Todorova T., Martinez R., Defeo E., Obregon P. (2009). Pectin activation of MAP kinase and gene expression is WAK2 dependent. Plant J..

[23] Engelsdorf T., Gigli-Bisceglia N., Veerabagu M., McKenna J.F., Vaahtera L., Augstein F., Does D., van der Zipfel C., Hamann T. (2018). The plant cell wall integrity maintenance and immune signaling systems cooperate to control stress responses in Arabidopsis thaliana. Sci. Signal..

[24] Hématy K., Sado P.-E., van Tuinen A., Rochange S., Desnos T., Balzergue S., Pelletier S., Renou J.-P., Höfte H. (2007). A Receptor-Like Kinase Mediates the Response of Arabidopsis Cells to the Inhibition of Cellulose Synthesis. Curr. Biol..

[25] Gonneau M., Desprez T., Martin M., Doblas V.G., Bacete L., Miart F., Sormani R., Hématy K., Renou J., Landrein B. (2018). Receptor Kinase THESEUS1 Is a Rapid Alkalinization Factor 34 Receptor in Arabidopsis. Curr. Biol..

[26] Guo H., Li L., Ye H., Yu X., Algreen A., Yin Y. (2009). Three related receptor-like kinases are required for optimal cell elongation in Arabidopsis thaliana. Proc. Natl. Acad. Sci. USA.

[27] Feng W., Kita D., Peaucelle A., Cartwright H.N., Doan V., Duan Q., Liu M.-C.C., Maman J., Steinhorst L., Schmitz-Thom I. (2018). The FERONIA Receptor Kinase Maintains Cell-Wall Integrity during Salt Stress through Ca^2+^ Signaling. Curr. Biol..

[28] Shih H.-W.W., Miller N.D., Dai C., Spalding E.P., Monshausen G.B. (2014). The receptor-like kinase FERONIA is required for mechanical signal transduction in Arabidopsis seedlings. Curr. Biol..

[29] Boisson-Dernier A., Lituiev D.S., Nestorova A., Franck C.M., Thirugnanarajah S., Grossniklaus U. (2013). ANXUR Receptor-Like Kinases Coordinate Cell Wall Integrity with Growth at the Pollen Tube Tip via NADPH Oxidases. PLoS Biol..

[30] Dünser K., Gupta S., Herger A., Feraru M.I., Ringli C., Kleine-Vehn J., Kleine-Vehn J. (2019). Extracellular matrix sensing by FERONIA and Leucine-Rich Repeat Extensins controls vacuolar expansion during cellular elongation in Arabidopsis thaliana. EMBO J..

[31] Herger A., Dünser K., Kleine-Vehn J., Ringli C. (2019). Leucine-Rich Repeat Extensin Proteins and Their Role in Cell Wall Sensing. Curr. Biol..

[32] Herger A., Gupta S., Kadler G., Franck C.M., Boisson-Dernier A., Ringli C. (2020). Overlapping functions and protein-protein interactions of LRR-extensins in Arabidopsis. PLoS Genet..

[33] Haffani Y.Z., Silva-Gagliardi N.F., Sewter S.K., Grace Aldea M., Zhao Z., Nakhamchik A., Cameron R.K., Goring D.R. (2006). Altered Expression of PERK Receptor Kinases in Arabidopsis Leads to Changes in Growth and Floral Organ Formation. Plant Signal. Behav..

[34] Humphrey T.V., Haasen K.E., Grace Aldea-Brydges M., Sun H., Zayed Y., Indriolo E., Goring D.R., Aldea-brydges M.G., Sun H., Zayed Y. (2015). PERK–KIPK–KCBP signalling negatively regulates root growth in Arabidopsis thaliana. J. Exp. Bot..

[35] Bai L., Zhang G., Zhou Y., Zhang Z., Wang W., Du Y., Wu Z., Song C.-P. (2009). Plasma membrane-associated proline-rich extensin-like receptor kinase 4, a novel regulator of Ca signalling, is required for abscisic acid responses in Arabidopsis thaliana. Plant J..

[36] Xu S.L., Rahman A., Baskin T.I., Kieber J.J. (2008). Two leucine-rich repeat receptor kinases mediate signaling, linking cell wall biosynthesis and ACC synthase in arabidopsis. Plant Cell.

[37] Shi H., Kim Y.S., Guo Y., Stevenson B., Zhu J.K. (2003). The Arabidopsis SOS5 locus encodes a putative cell surface adhesion protein and is required for normal cell expansion. Plant Cell.

[38] Seifert G.J., Xue H., Acet T. (2014). The Arabidopsis thaliana FASCICLIN LIKE ARABINOGALACTAN PROTEIN 4 gene acts synergistically with abscisic acid signalling to control root growth. Ann. Bot..

[39] Van der Does D., Boutrot F., Engelsdorf T., Rhodes J., McKenna J.F., Vernhettes S., Koevoets I., Tintor N., Veerabagu M., Miedes E. (2017). The Arabidopsis leucine-rich repeat receptor kinase MIK2/LRR-KISS connects cell wall integrity sensing, root growth and response to abiotic and biotic stresses. PLoS Genet..

[40] Chaudhary A., Chen X., Gao J., Leśniewska B., Hammerl R., Dawid C., Schneitz K. (2020). The Arabidopsis receptor kinase STRUBBELIG regulates the response to cellulose deficiency. PLoS Genet..

[41] Martinac B., Buechner M., Delcour A.H., Adler J., Kung C. (1987). Pressure-sensitive ion channel in Escherichia coli. Proc. Natl. Acad. Sci. USA.

[42] Schleyer M., Schmid R., Bakker E.P. (1993). Transient, specific and extremely rapid release of osmolytes from growing cells of Escherichia coli K-12 exposed to hypoosmotic shock. Arch. Microbiol..

[43] Booth I.R., Louis P. (1999). Managing hypoosmotic stress: Aquaporins and medianosensitive channels in Escherichia coli. Curr. Opin. Microbiol..

[44] Levina N. (1999). Protection of Escherichia coli cells against extreme turgor by activation of MscS and MscL mechanosensitive channels: Identification of genes required for MscS activity. EMBO J..

[45] Berrier C., Besnard M., Ajouz B., Coulombe A., Ghazi A. (1996). Multiple Mechanosensitive Ion Channels from Escherichia coli, Activated at Different Thresholds of Applied Pressure. J. Membr. Biol..

[46] Blount P., Schroeder M.J., Kung C. (1997). Mutations in a bacterial mechanosensitive channel change the cellular response to osmotic stress. J. Biol. Chem..

[47] Chang G., Spencer R.H., Lee A.T., Barclay M.T., Rees D.C. (1998). Structure of the MscL Homolog from Mycobacterium tuberculosis: A Gated Mechanosensitive Ion Channel. Science.

[48] Sukharev S. (2002). Purification of the Small Mechanosensitive Channel of Escherichia coli (MscS): The Subunit Structure, Conduction, and Gating Characteristicsin Liposomes. Biophys. J..

[49] Jiao R., Cui D., Wang S.C., Li D., Wang Y.F. (2017). Interactions of the mechanosensitive channels with extracellular matrix, integrins, and cytoskeletal network in osmosensation. Front. Mol. Neurosci..

[50] Sukharev S.I., Blount P., Martinac B., Blattner F.R., Kung C. (1994). A large-conductance mechanosensitive channel in *E. coli* encoded by mscL alone. Nature.

[51] Ernstrom G.G., Chalfie M. (2002). Genetics of sensory mechanotransduction. Annu. Rev. Genet..

[52] Delmas P., Coste B. (2013). Mechano-gated ion channels in sensory systems. Cell.

[53] Jin P., Jan L.Y., Jan Y.-N. (2020). Mechanosensitive Ion Channels: Structural Features Relevant to Mechanotransduction Mechanisms. Annu. Rev. Neurosci..

[54] Chalfie M., Sulston J. (1981). Developmental genetics of the mechanosensory neurons of Caenorhabditis elegans. Dev. Biol..

[55] O’Hagan R., Chalfie M., Goodman M.B. (2005). The MEC-4 DEG/ENaC channel of Caenorhabditis elegans touch receptor neurons transduces mechanical signals. Nat. Neurosci..

[56] Brown A.L., Liao Z., Goodman M.B. (2008). MEC-2 and MEC-6 in the Caenorhabditis elegans sensory mechanotransduction complex: Auxiliary subunits that enable channel activity. J. Gen. Physiol..

[57] Chelur D.S., Ernstrom G.G., Goodman M.B., Yao C.A., Chen L., O’Hagan R., Chalfie M. (2002). The mechanosensory protein MEC-6 is a subunit of the C. elegans touch-cell degenerin channel. Nature.

[58] Goodman M.B., Ernstrom G.G., Chelur D.S., O’Hagan R., Yao C.A., Chalfie M. (2002). MEC-2 regulates C. elegans DEG/ENaC channels needed for mechanosensation. Nature.

[59] Batiza A.F., Schulz T., Masson P.H. (1996). Yeast Respond to Hypotonic Shock with a Calcium Pulse. J. Biol. Chem..

[60] Matsumoto T.K., Ellsmore A.J., Cessna S.G., Low P.S., Pardo J.M.J.M., Bressan R.A., Hasegawa P.M. (2002). An Osmotically Induced Cytosolic Ca^2+^ Transient Activates Calcineurin Signaling to Mediate Ion Homeostasis and Salt Tolerance of Saccharomyces cerevisiae. J. Biol. Chem..

[61] Fischer M., Schnell N., Chattaway J., Davies P., Dixon G., Sanders D. (1997). The Saccharomyces cerevisiae CCH1 gene is involved in calcium influx and mating. FEBS Lett..

[62] Paidhungat M., Garrett S. (1997). A homolog of mammalian, voltage-gated calcium channels mediates yeast pheromone-stimulated Ca^2+^ uptake and exacerbates the cdc1(Ts) growth defect. Mol. Cell. Biol..

[63] Yoshimura H., Tada T., Iida H. (2004). Subcellular localization and oligomeric structure of the yeast putative stretch-activated Ca^2+^ channel component Mid1. Exp. Cell Res..

[64] Iida H., Nakamura H., Ono T., Okumura M.S., Anraku Y. (1994). MID1, a novel Saccharomyces cerevisiae gene encoding a plasma membrane protein, is required for Ca^2+^ influx and mating. Mol. Cell. Biol..

[65] Locke E.G., Bonilla M., Liang L., Takita Y., Cunningham K.W. (2000). A Homolog of Voltage-Gated Ca^2+^ Channels Stimulated by Depletion of Secretory Ca^2+^ in Yeast. Mol. Cell. Biol..

[66] Peiter E., Fischer M., Sidaway K., Roberts S.K., Sanders D. (2005). The Saccharomyces cerevisiae Ca^2+^ channel Cch1pMid1p is essential for tolerance to cold stress and iron toxicity. FEBS Lett..

[67] Cyert M.S. (2003). Calcineurin signaling in Saccharomyces cerevisiae: How yeast go crazy in response to stress. Biochem. Biophys. Res. Commun..

[68] Stathopoulos A.M., Cyert M.S. (1997). Calcineurin acts through the CRZ1/TCN1-encoded transcription factor to regulate gene expression in yeast. Genes Dev..

[69] Matheos D.P., Kingsbury T.J., Ahsan U.S., Cunningham K.W. (1997). Tcn1p/Crz1p, a calcineurin-dependent transcription factor that differentially regulates gene expression in Saccharomyces cerevisiae. Genes Dev..

[70] Garrett-Engele P., Moilanen B., Cyert M.S. (1995). Calcineurin, the Ca2+/calmodulin-dependent protein phosphatase, is essential in yeast mutants with cell integrity defects and in mutants that lack a functional vacuolar H(+)-ATPase. Mol. Cell. Biol..

[71] Zhao C., Jung U.S., Garrett-Engele P., Roe T., Cyert M.S., Levin D.E. (1998). Temperature-induced expression of yeast FKS2 is under the dual control of protein kinase C and calcineurin. Mol. Cell. Biol..

[72] Levin D.E. (2011). Regulation of cell wall biogenesis in Saccharomyces cerevisiae: The cell wall integrity signaling pathway. Genetics.

[73] Heinisch J.J., Dupres V., Wilk S., Jendretzki A., Dufrêne Y.F. (2010). Single-molecule atomic force microscopy reveals clustering of the yeast plasma-membrane sensor Wsc1. PLoS ONE.

[74] Kechagia J.Z., Ivaska J., Roca-Cusachs P. (2019). Integrins as biomechanical sensors of the microenvironment. Nat. Rev. Mol. Cell Biol..

[75] Saini K., Discher D.E. (2019). Forced Unfolding of Proteins Directs Biochemical Cascades. Biochemistry.

[76] Hynes R.O. (2002). Integrins: Bidirectional, allosteric signaling machines. Cell.

[77] Hynes R.O. (2004). The emergence of integrins: A personal and historical perspective. Matrix Biol..

[78] Takagi J., Petre B.M., Walz T., Springer T.A. (2002). Global conformational rearrangements in integrin extracellular domains in outside-in and inside-out signaling. Cell.

[79] Humphries J.D., Byron A., Humphries M.J. (2006). Integrin ligands at a glance. J. Cell Sci..

[80] Puklin-Faucher E., Vogel V. (2009). Integrin activation dynamics between the RGD-binding site and the headpiece hinge. J. Biol. Chem..

[81] Zhu J., Luo B.-H., Xiao T., Zhang C., Nishida N., Springer T.A. (2008). Structure of a complete integrin ectodomain in a physiologic resting state and activation and deactivation by applied forces. Mol. Cell.

[82] Chen W., Lou J., Hsin J., Schulten K., Harvey S.C., Zhu C. (2011). Molecular dynamics simulations of forced unbending of integrin α(v)β₃. PLoS Comput. Biol..

[83] Nordenfelt P., Elliott H.L., Springer T.A. (2016). Coordinated integrin activation by actin-dependent force during T-cell migration. Nat. Commun..

[84] Burridge K. (2017). Focal adhesions: A personal perspective on a half century of progress. FEBS J..

[85] Calderwood D.A., Campbell I.D., Critchley D.R. (2013). Talins and kindlins: Partners in integrin-mediated adhesion. Nat. Rev. Mol. Cell Biol..

[86] Case L.B., Baird M.A., Shtengel G., Campbell S.L., Hess H.F., Davidson M.W., Waterman C.M. (2015). Molecular mechanism of vinculin activation and nanoscale spatial organization in focal adhesions. Nat. Cell Biol..

[87] Elosegui-Artola A., Trepat X., Roca-Cusachs P. (2018). Control of Mechanotransduction by Molecular Clutch Dynamics. Trends Cell Biol..

[88] Hamant O., Inoue D., Bouchez D., Dumais J., Mjolsness E. (2019). Are microtubules tension sensors?. Nat. Commun..

[89] Fruleux A., Verger S., Boudaoud A. (2019). Feeling stressed or strained? A biophysical model for cell wall mechanosensing in plants. Front. Plant Sci..

[90] Bringmann M., Bergmann D.C. (2017). Tissue-Wide Mechanical Forces Influence the Polarity of Stomatal Stem Cells in Arabidopsis. Curr. Biol..

[91] Bidhendi A.J., Altartouri B., Gosselin F.P., Geitmann A. (2019). Mechanical Stress Initiates and Sustains the Morphogenesis of Wavy Leaf Epidermal Cells. Cell Rep..

[92] Lin W., Yang Z. (2020). Unlocking the mechanisms behind the formation of interlocking pavement cells. Curr. Opin. Plant Biol..

[93] Sampathkumar A. (2020). Mechanical feedback-loop regulation of morphogenesis in plants. Development.

[94] Kopecká M., Gabriel M. (1992). The influence of congo red on the cell wall and (1—3)-beta-D-glucan microfibril biogenesis in Saccharomyces cerevisiae. Arch. Microbiol..

[95] Ketela T., Green R., Bussey H. (1999). Saccharomyces cerevisiae mid2p is a potential cell wall stress sensor and upstream activator of the PKC1-MPK1 cell integrity pathway. J. Bacteriol..

[96] Nobel H., de Ruiz C., Martin H., Morris W., Brul S., Molina M., Klis F.M. (2000). Cell wall perturbation in yeast results in dual phosphorylation of the Slt2/Mpk1 MAP kinase and in an Slt2-mediated increase in FKS2-lacZ expression, glucanase resistance and thermotolerance. Microbiology.

[97] Martín H., Rodríguez-Pachón J.M., Ruiz C., Nombela C., Molina M. (2000). Regulatory mechanisms for modulation of signaling through the cell integrity Slt2-mediated pathway in Saccharomyces cerevisiae. J. Biol. Chem..

[98] Jung U.S., Sobering A.K., Romeo M.J., Levin D.E. (2002). Regulation of the yeast Rlm1 transcription factor by the Mpk1 cell wall integrity MAP kinase. Mol. Microbiol..

[99] Reinoso-Martín C., Schüller C., Schuetzer-Muehlbauer M., Kuchler K. (2003). The yeast protein kinase C cell integrity pathway mediates tolerance to the antifungal drug caspofungin through activation of Slt2p mitogen-activated protein kinase signaling. Eukaryot. Cell.

[100] García R., Bermejo C., Grau C., Pérez R., Rodríguez-Peña J.M., Francois J., Nombela C., Arroyo J. (2004). The global transcriptional response to transient cell wall damage in Saccharomyces cerevisiae and its regulation by the cell integrity signaling pathway. J. Biol. Chem..

[101] García R., Rodríguez-Peña J.M., Bermejo C., Nombela C., Arroyo J. (2009). The high osmotic response and cell wall integrity pathways cooperate to regulate transcriptional responses to zymolyase-induced cell wall stress in Saccharomyces cerevisiae. J. Biol. Chem..

[102] Kuranda K., Leberre V., Sokol S., Palamarczyk G., François J. (2006). Investigating the caffeine effects in the yeast Saccharomyces cerevisiae brings new insights into the connection between TOR, PKC and Ras/cAMP signalling pathways. Mol. Microbiol..

[103] Arroyo J., Bermejo C., García R., Rodríguez-Peña J.M. (2009). Genomics in the detection of damage in microbial systems: Cell wall stress in yeast. Clin. Microbiol. Infect..

[104] Terashima H., Yabuki N., Arisawa M., Hamada K., Kitada K. (2000). Up-regulation of genes encoding glycosylphosphatidylinositol (GPI)-attached proteins in response to cell wall damage caused by disruption of FKS1 in Saccharomyces cerevisiae. Mol. Gen. Genet..

[105] Bulik D.A., Olczak M., Lucero H.A., Osmond B.C., Robbins P.W., Specht C.A. (2003). Chitin synthesis in Saccharomyces cerevisiae in response to supplementation of growth medium with glucosamine and cell wall stress. Eukaryot. Cell.

[106] Lagorce A., Hauser N.C., Labourdette D., Rodriguez C., Martin-Yken H., Arroyo J., Hoheisel J.D., François J. (2003). Genome-wide analysis of the response to cell wall mutations in the yeast Saccharomyces cerevisiae. J. Biol. Chem..

[107] Verna J., Lodder A., Lee K., Vagts A., Ballester R. (1997). A family of genes required for maintenance of cell wall integrity and for the stress response in Saccharomyces cerevisiae. Proc. Natl. Acad. Sci. USA.

[108] Gray J.V., Ogas J.P., Kamada Y., Stone M., Levin D.E., Herskowitz I. (1997). A role for the Pkc1 MAP kinase pathway of Saccharomyces cerevisiae in bud emergence and identification of a putative upstream regulator. EMBO J..

[109] Rajavel M., Philip B., Buehrer B.M., Errede B., Levin D.E. (1999). Mid2 is a putative sensor for cell integrity signaling in Saccharomyces cerevisiae. Mol. Cell. Biol..

[110] Dupres V., Alsteens D., Wilk S., Hansen B., Heinisch J.J., Dufrêne Y.F. (2009). The yeast Wsc1 cell surface sensor behaves like a nanospring in vivo. Nat. Chem. Biol..

[111] Neeli-Venkata R., Celador R., Sanchez Y., Minc N. (2020). Detection of Surface Forces by a Cell Wall Mechanosensor. bioRxiv.

[112] Kock C., Dufrêne Y.F., Heinisch J.J. (2015). Up against the wall: Is yeast cell wall integrity ensured by mechanosensing in plasma membrane microdomains?. Appl. Environ. Microbiol..

[113] Rodicio R., Heinisch J.J. (2010). Together we are strong—Cell wall integrity sensors in yeasts. Yeast.

[114] Levin D.E. (2005). Cell Wall Integrity Signaling in *Saccharomyces cerevisiae*. Microbiol. Mol. Biol. Rev..

[115] Sanz A.B., García R., Rodríguez-Peña J.M., Arroyo J. (2018). The CWI pathway: Regulation of the transcriptional adaptive response to cell wall stress in yeast. J. Fungi.

[116] Gualtieri T., Ragni E., Mizzi L., Fascio U., Popolo L. (2004). The cell wall sensor Wsc1p is involved in reorganization of actin cytoskeleton in response to hypo-osmotic shock in Saccharomyces cerevisiae. Yeast.

[117] Ueno K., Saito M., Nagashima M., Kojima A., Nishinoaki S., Toshima J.Y., Toshima J. (2014). V-ATPase-dependent luminal acidification is required for endocytic recycling of a yeast cell wall stress sensor, Wsc1p. Biochem. Biophys. Res. Commun..

[118] Mao K., Klionsky D.J. (2011). MAPKs regulate mitophagy in Saccharomyces cerevisiae. Autophagy.

[119] Jacobs M. (1954). The effect of wind sway on the form and development of Pinus radiata D. Don. Aust. J. Bot..

[120] Turgeon R., Webb J.A. (1971). Growth inhibition by mechanical stress. Science.

[121] Jaffe M.J. (1973). Thigmomorphogenesis: The response of plant growth and development to mechanical stimulation: With special reference to Bryonia dioica. Planta.

[122] Braam J., Davis R.W. (1990). Rain-, wind-, and touch-induced expression of calmodulin and calmodulin-related genes in Arabidopsis. Cell.

[123] Lee D., Polisensky D.H., Braam J. (2005). Genome-wide identification of touch- and darkness-regulated Arabidopsis genes: A focus on calmodulin-like and XTH genes. New Phytol..

[124] Fagard M., Desnos T., Desprez T., Goubet F., Refregier G., Mouille G., McCann M., Rayon C., Vernhettes S., Höfte H. (2000). PROCUSTE1 encodes a cellulose synthase required for normal cell elongation specifically in roots and dark-grown hypocotyls of Arabidopsis. Plant Cell.

[125] Ellis C., Turner J.G. (2001). The Arabidopsis Mutant cev1 Has Constitutively Active Jasmonate and Ethylene Signal Pathways and Enhanced Resistance to Pathogens. Plant Cell.

[126] Ellis C., Karafyllidis I., Wasternack C., Turner J.G. (2002). The Arabidopsis Mutant cev1 Links Cell Wall Signaling to Jasmonate and Ethylene Responses. Plant Cell.

[127] Caño-Delgado A., Penfield S., Smith C., Catley M., Bevan M., Cano-Delgado A., Penfield S., Smith C., Catley M., Bevan M. (2003). Reduced cellulose synthesis invokes lignification and defense responses in Arabidopsis thaliana. Plant J..

[128] Pagant S., Bichet A., Sugimoto K., Lerouxel O., Desprez T., McCann M., Lerouge P., Vernhettes S., Höfte H. (2002). KOBITO1 encodes a novel plasma membrane protein necessary for normal synthesis of cellulose during cell expansion in Arabidopsis. Plant Cell.

[129] Brocard-Gifford I., Lynch T.J., Garcia M.E., Malhotra B., Finkelstein R.R. (2004). The Arabidopsis thaliana ABSCISIC ACID-INSENSITIVE8 encodes a novel protein mediating abscisic acid and sugar responses essential for growth. Plant Cell.

[130] Chen Z., Hong X., Zhang H., Wang Y., Li X., Zhu J.-K., Gong Z. (2005). Disruption of the cellulose synthase gene, AtCesA8/IRX1, enhances drought and osmotic stress tolerance in Arabidopsis. Plant J..

[131] Hernández-Blanco C., Feng D.X., Hu J., Sánchez-Vallet A., Deslandes L., Llorente F., Berrocal-Lobo M., Keller H., Barlet X., Sánchez-Rodríguez C. (2007). Impairment of cellulose synthases required for Arabidopsis secondary cell wall formation enhances disease resistance. Plant Cell.

[132] Wormit A., Butt S.M., Chairam I., McKenna J.F., Nunes-Nesi A., Kjaer L., O’Donnelly K., Fernie A.R., Woscholski R., Barter M.C.L. (2012). Osmosensitive Changes of Carbohydrate Metabolism in Response to Cellulose Biosynthesis Inhibition. Plant Physiol..

[133] Hamann T. (2015). The plant cell wall integrity maintenance mechanism—Concepts for organization and mode of action. Plant Cell Physiol..

[134] Engelsdorf T., Kjaer L., Gigli-Bisceglia N., Vaahtera L., Bauer S., Miedes E., Wormit A., James L., Chairam I., Molina A. (2019). Functional characterization of genes mediating cell wall metabolism and responses to plant cell wall integrity impairment. BMC Plant Biol..

[135] Wilson M.E., Maksaev G., Haswell E.S. (2013). MscS-like mechanosensitive channels in plants and microbes. Biochemistry.

[136] Li Y., Hu Y., Wang J., Liu X., Zhang W., Sun L. (2020). Structural Insights into a Plant Mechanosensitive Ion Channel MSL1. Cell Rep..

[137] Hamilton E.S., Schlegel A.M., Haswell E.S. (2015). United in diversity: Mechanosensitive ion channels in plants. Annu. Rev. Plant Biol..

[138] Haswell E.S., Meyerowitz E.M. (2006). MscS-like proteins control plastid size and shape in Arabidopsis thaliana. Curr. Biol..

[139] Hamilton E.S., Jensen G.S., Maksaev G., Katims A., Sherp A.M., Haswell E.S. (2015). Mechanosensitive channel MSL8 regulates osmotic forces during pollen hydration and germination. Science.

[140] Peyronnet R., Haswell E.S., Barbier-Brygoo H., Frachisse J.-M. (2008). AtMSL9 and AtMSL10: Sensors of plasma membrane tension in Arabidopsis roots. Plant Signal. Behav..

[141] Veley K.M., Maksaev G., Frick E.M., January E., Kloepper S.C., Haswell E.S. (2014). Arabidopsis MSL10 has a regulated cell death signaling activity that is separable from its mechanosensitive ion channel activity. Plant Cell.

[142] Zou Y., Chintamanani S., He P., Fukushige H., Yu L., Shao M., Zhu L., Hildebrand D.F., Tang X., Zhou J.-M. (2016). A gain-of-function mutation in Msl10 triggers cell death and wound-induced hyperaccumulation of jasmonic acid in Arabidopsis. J. Integr. Plant Biol..

[143] Zhang Z., Tateda C., Jiang S.-C., Shrestha J., Jelenska J., Speed D.J., Greenberg J.T. (2017). A Suite of Receptor-Like Kinases and a Putative Mechano-Sensitive Channel Are Involved in Autoimmunity and Plasma Membrane-Based Defenses in Arabidopsis. Mol. Plant Microbe. Interact..

[144] Kohorn B.D., Hoon D., Minkoff B.B., Sussman M.R., Kohorn S.L. (2016). Rapid Oligo-Galacturonide Induced Changes in Protein Phosphorylation in Arabidopsis. Mol. Cell. Proteom..

[145] Nakano M., Iida K., Nyunoya H., Iida H. (2011). Determination of Structural Regions Important for Ca^2+^ Uptake Activity in Arabidopsis MCA1 and MCA2 Expressed in Yeast. Plant Cell Physiol..

[146] Kamano S., Kume S., Iida K., Lei K.-J.J., Nakano M., Nakayama Y., Iida H. (2015). Transmembrane Topologies of Ca^2+^-permeable Mechanosensitive Channels MCA1 and MCA2 in Arabidopsis thaliana. J. Biol. Chem..

[147] Shigematsu H., Iida K., Nakano M., Chaudhuri P., Iida H., Nagayama K. (2014). Structural characterization of the mechanosensitive channel candidate MCA2 from Arabidopsis thaliana. PLoS ONE.

[148] Furuichi T., Iida H., Sokabe M., Tatsumi H. (2012). Expression of Arabidopsis MCA1 enhanced mechanosensitive channel activity in the Xenopus laevis oocyte plasma membrane. Plant Signal. Behav..

[149] Mori K., Renhu N., Naito M., Nakamura A., Shiba H., Yamamoto T., Suzaki T., Iida H., Miura K. (2018). Ca^2+^-permeable mechanosensitive channels MCA1 and MCA2 mediate cold-induced cytosolic Ca^2+^ increase and cold tolerance in Arabidopsis. Sci. Rep..

[150] Hattori T., Otomi Y., Nakajima Y., Soga K., Wakabayashi K., Iida H., Hoson T. (2020). MCA1 and MCA2 Are Involved in the Response to Hypergravity in Arabidopsis Hypocotyls. Plants.

[151] Nakano M., Furuichi T., Sokabe M., Iida H., Tatsumi H. (2021). The gravistimulation-induced very slow Ca^2+^ increase in Arabidopsis seedlings requires MCA1, a Ca^2+^-permeable mechanosensitive channel. Sci. Rep..

[152] Iida H., Furuichi T., Nakano M., Toyota M., Sokabe M., Tatsumi H. (2014). New candidates for mechano-sensitive channels potentially involved in gravity sensing in Arabidopsis thaliana. Plant Biol..

[153] Murthy S.E., Dubin A.E., Whitwam T., Jojoa-Cruz S., Cahalan S.M., Mousavi S.A.R., Ward A.B., Patapoutian A. (2018). OSCA/TMEM63 are an Evolutionarily Conserved Family of Mechanically Activated Ion Channels. eLife.

[154] Zhang M., Wang D., Kang Y., Wu J.-X., Yao F., Pan C., Yan Z., Song C., Chen L. (2018). Structure of the mechanosensitive OSCA channels. Nat. Struct. Mol. Biol..

[155] Kiyosue T., Yamaguchi-Shinozaki K., Shinozaki K. (1994). Cloning of cDNAs for genes that are early-responsive to dehydration stress (ERDs) in Arabidopsis thaliana L.: Identification of three ERDs as HSP cognate genes. Plant Mol. Biol..

[156] Meinke D.W. (2020). Genome-wide identification of EMBRYO-DEFECTIVE (EMB) genes required for growth and development in Arabidopsis. New Phytol..

[157] Liang Z., Demko V., Wilson R.C., Johnson K.A., Ahmad R., Perroud P.-F., Quatrano R., Zhao S., Shalchian-Tabrizi K., Otegui M.S. (2013). The catalytic domain CysPc of the DEK1 calpain is functionally conserved in land plants. Plant J..

[158] Johnson K.L., Faulkner C., Jeffree C.E., Ingram G.C. (2008). The phytocalpain defective kernel 1 is a novel Arabidopsis growth regulator whose activity is regulated by proteolytic processing. Plant Cell.

[159] Johnson K.L., Degnan K.A., Ross Walker J., Ingram G.C. (2005). AtDEK1 is essential for specification of embryonic epidermal cell fate. Plant J..

[160] Galletti R., Johnson K.L., Scofield S., San-Bento R., Watt A.M., Murray J.A.H., Ingram G.C. (2015). DEFECTIVE KERNEL 1 promotes and maintains plant epidermal differentiation. Development.

[161] Lid S.E., Olsen L., Nestestog R., Aukerman M., Brown R.C., Lemmon B., Mucha M., Opsahl-Sorteberg H.-G., Olsen O.-A. (2005). Mutation in the Arabidopisis thaliana DEK1 calpain gene perturbs endosperm and embryo development while over-expression affects organ development globally. Planta.

[162] Liang Z., Brown R.C., Fletcher J.C., Opsahl-Sorteberg H.-G. (2015). Calpain-Mediated Positional Information Directs Cell Wall Orientation to Sustain Plant Stem Cell Activity, Growth and Development. Plant Cell Physiol..

[163] Amanda D., Doblin M.S., MacMillan C.P., Galletti R., Golz J.F., Bacic A., Ingram G.C., Johnson K.L. (2017). Arabidopsis DEFECTIVE KERNEL1 regulates cell wall composition and axial growth in the inflorescence stem. Plant Direct.

[164] Franco S.J., Huttenlocher A. (2005). Regulating cell migration: Calpains make the cut. J. Cell Sci..

[165] Croall D.E., Ersfeld K. (2007). The calpains: Modular designs and functional diversity. Genome Biol..

[166] Barzegari A., Omidi Y., Ostadrahimi A., Gueguen V., Meddahi-Pellé A., Nouri M., Pavon-Djavid G. (2020). The role of Piezo proteins and cellular mechanosensing in tuning the fate of transplanted stem cells. Cell Tissue Res..

[167] Bengough A.G., McKenzie B.M., Hallett P.D., Valentine T.A. (2011). Root elongation, water stress, and mechanical impedance: A review of limiting stresses and beneficial root tip traits. J. Exp. Bot..

[168] Hecht K. (1912). Studien über den Vorgang der Plasmolyse. Beitr. Biol. Pflanz..

[169] Lang I., Barton D.A., Overall R.L. (2004). Membrane-wall attachments in plasmolysed plant cells. Protoplasma.

[170] Liu Z., Persson S., Sánchez-Rodríguez C. (2015). At the border: The plasma membrane-cell wall continuum. J. Exp. Bot..

[171] Knepper C., Savory E.A., Day B. (2011). Arabidopsis NDR1 is an integrin-like protein with a role in fluid loss and plasma membrane-cell wall adhesion. Plant Physiol..

[172] Delley P.-A., Hall M.N. (1999). Cell Wall Stress Depolarizes Cell Growth via Hyperactivation of Rho1. J. Cell Biol..

[173] Schoenwaelder S.M., Burridge K. (1999). Bidirectional signaling between the cytoskeleton and integrins. Curr. Opin. Cell Biol..

[174] He Z.-H., Fujiki M., Kohorn B.D. (1996). A Cell Wall-Associated, Receptor-Like Protein Kinase. J. Biol. Chem..

[175] Anderson C.M., Wagner T.A., Perret M., He Z.H., He D., Kohorn B.D. (2001). WAKs: Cell wall-associated kinases linking the cytoplasm to the extracellular matrix. Plant Mol. Biol..

[176] He Z.H., Cheeseman I., He D., Kohorn B.D. (1999). A cluster of five cell wall-associated receptor kinase genes, Wak1-5, are expressed in specific organs of Arabidopsis. Plant Mol. Biol..

[177] Decreux A., Messiaen J. (2005). Wall-associated kinase WAK1 interacts with cell wall pectins in a calcium-induced conformation. Plant Cell Physiol..

[178] Denoux C., Galletti R., Mammarella N., Gopalan S., Werck D., Lorenzo G., de Ferrari S., Ausubel F.M., Dewdney J. (2008). Activation of defense response pathways by OGs and Flg22 elicitors in Arabidopsis seedlings. Mol. Plant.

[179] Lorrai R., Ferrari S. (2021). Host Cell Wall Damage during Pathogen Infection: Mechanisms of Perception and Role in Plant-Pathogen Interactions. Plants.

[180] He Z.H., He D., Kohorn B.D. (1998). Requirement for the induced expression of a cell wall associated receptor kinase for survival during the pathogen response. Plant J..

[181] Wagner T.A., Kohorn B.D. (2001). Wall-associated kinases are expressed throughout plant development and are required for cell expansion. Plant Cell.

[182] Park A.R., Cho S.K., Yun U.J., Jin M.Y., Lee S.H., Sachetto-Martins G., Park O.K. (2001). Interaction of the Arabidopsis receptor protein kinase Wak1 with a glycine-rich protein, AtGRP-3. J. Biol. Chem..

[183] Kohorn B.D., Greed B.E., Mouille G., Verger S., Kohorn S.L. (2021). Effects of Arabidopsis wall associated kinase mutations on ESMERALDA1 and elicitor induced ROS. PLoS ONE.

[184] Franck C.M., Westermann J., Boisson-Dernier A. (2018). Plant Malectin-Like Receptor Kinases: From Cell Wall Integrity to Immunity and Beyond. Annu. Rev. Plant Biol..

[185] Desnos T., Orbović V., Bellini C., Kronenberger J., Caboche M., Traas J., Höfte H. (1996). Procuste1 mutants identify two distinct genetic pathways controlling hypocotyl cell elongation, respectively in dark- and light-grown Arabidopsis seedlings. Development.

[186] Desprez T. (2002). Resistance against Herbicide Isoxaben and Cellulose Deficiency Caused by Distinct Mutations in Same Cellulose Synthase Isoform CESA6. Plant Physiol..

[187] Merz D., Richter J., Gonneau M., Sanchez-Rodriguez C., Eder T., Sormani R., Martin M., Hématy K., Höfte H., Hauser M.-T.T. (2017). T-DNA alleles of the receptor kinase THESEUS1 with opposing effects on cell wall integrity signaling. J. Exp. Bot..

[188] Qu S., Zhang X., Song Y., Lin J., Shan X. (2017). THESEUS1 positively modulates plant defense responses against Botrytis cinerea through GUANINE EXCHANGE FACTOR4 signaling. J. Integr. Plant Biol..

[189] Murphy E., Smet I. (2014). De Understanding the RALF family: A tale of many species. Trends Plant Sci..

[190] Blackburn M.R., Haruta M., Moura D.S. (2020). Twenty Years of Progress in Physiological and Biochemical Investigation of RALF Peptides. Plant Physiol..

[191] Campbell L., Turner S.R. (2017). A Comprehensive Analysis of RALF Proteins in Green Plants Suggests There Are Two Distinct Functional Groups. Front. Plant Sci..

[192] Morato do Canto A., Ceciliato P.H.O., Ribeiro B., Ortiz Morea F.A., Franco Garcia A.A., Silva-Filho M.C., Moura D.S. (2014). Biological activity of nine recombinant AtRALF peptides: Implications for their perception and function in Arabidopsis. Plant Physiol. Biochem..

[193] Haruta M., Sabat G., Stecker K., Minkoff B.B., Sussman M.R. (2014). A peptide hormone and its receptor protein kinase regulate plant cell expansion. Science.

[194] Kessler S.A., Lindner H., Jones D.S., Grossniklaus U. (2015). Functional analysis of related CrRLK1L receptor-like kinases in pollen tube reception. EMBO Rep..

[195] Westermann J., Streubel S., Franck C.M., Lentz R., Dolan L., Boisson-Dernier A. (2019). An Evolutionarily Conserved Receptor-Like Kinases Signaling Module Controls Cell Wall Integrity during Tip Growth. Curr. Biol..

[196] Zhu S., Martínez Pacheco J., Estevez J.M., Yu F. (2020). Autocrine regulation of root hair size by the RALF-FERONIA-RSL4 signaling pathway. New Phytol..

[197] Kim D., Yang J., Gu F., Park S., Combs J., Adams A., Mayes H.B., Jeon S.J., Bahk J.D., Nielsen E. (2021). A temperature-sensitive FERONIA mutant allele that alters root hair growth. Plant Physiol..

[198] Guo H., Nolan T.M., Song G., Liu S., Xie Z., Chen J., Schnable P.S., Walley J.W., Yin Y. (2018). FERONIA Receptor Kinase Contributes to Plant Immunity by Suppressing Jasmonic Acid Signaling in Arabidopsis thaliana. Curr. Biol..

[199] Huang L., Li X., Zhang W., Ung N., Liu N., Yin X., Li Y., McEwan R.E., Dilkes B., Dai M. (2020). Endosidin20 targets the cellulose synthase catalytic domain to inhibit cellulose biosynthesis. Plant Cell.

[200] Song Y., Wilson A.J., Zhang X.-C., Thoms D., Sohrabi R., Song S., Geissmann Q., Liu Y., Walgren L., He S.Y. (2021). FERONIA restricts Pseudomonas in the rhizosphere microbiome via regulation of reactive oxygen species. Nat. Plants.

[201] Zhao C., Zayed O., Yu Z., Jiang W., Zhu P., Hsu C.-C.C., Zhang L., Tao W.A., Lozano-Durán R., Zhu J.-K.K. (2018). Leucine-rich repeat extensin proteins regulate plant salt tolerance in Arabidopsis. Proc. Natl. Acad. Sci. USA.

[202] Barbez E., Dünser K., Gaidora A., Lendl T., Busch W. (2017). Auxin steers root cell expansion via apoplastic pH regulation in Arabidopsis thaliana. Proc. Natl. Acad. Sci. USA.

[203] Doblas V.G., Gonneau M., Höfte H. (2018). Cell wall integrity signaling in plants: Malectin-domain kinases and lessons from other kingdoms. Cell Surf..

[204] Stegmann M., Monaghan J., Smakowska-Luzan E., Rovenich H., Lehner A., Holton N., Belkhadir Y., Zipfel C. (2017). The receptor kinase FER is a RALF-regulated scaffold controlling plant immune signaling. Science.

[205] Gjetting S.K., Mahmood K., Shabala L., Kristensen A., Shabala S., Palmgren M., Fuglsang A.T. (2020). Evidence for multiple receptors mediating RALF-triggered Ca^2+^ signaling and proton pump inhibition. Plant J..

[206] Li C., Yeh F.-L., Cheung A.Y., Duan Q., Kita D., Liu M.-C., Maman J., Luu E.J., Wu B.W., Gates L. (2015). Glycosylphosphatidylinositol-anchored proteins as chaperones and co-receptors for FERONIA receptor kinase signaling in Arabidopsis. eLife.

[207] Yu Y., Chakravorty D., Assmann S.M. (2018). The G Protein β-Subunit, AGB1, Interacts with FERONIA in RALF1-Regulated Stomatal Movement. Plant Physiol..

[208] Yu Y., Assmann S.M. (2018). Inter-relationships between the heterotrimeric Gβ subunit AGB1, the receptor-like kinase FERONIA, and RALF1 in salinity response. Plant Cell Environ..

[209] Yu M., Li R., Cui Y., Chen W., Li B., Zhang X., Bu Y., Cao Y., Xing J., Jewaria P.K. (2020). The RALF1-FERONIA interaction modulates endocytosis to mediate control of root growth in Arabidopsis. Development.

[210] Zhu S., Estévez J.M., Liao H., Zhu Y., Yang T., Li C., Wang Y., Li L., Liu X., Pacheco J.M. (2020). The RALF1-FERONIA Complex Phosphorylates eIF4E1 to Promote Protein Synthesis and Polar Root Hair Growth. Mol. Plant.

[211] Wang L., Yang T., Wang B., Lin Q., Zhu S., Li C., Ma Y., Tang J., Xing J., Li X. (2020). RALF1-FERONIA complex affects splicing dynamics to modulate stress responses and growth in plants. Sci. Adv..

[212] Mang H., Feng B., Hu Z., Boisson-Dernier A., Franck C.M., Meng X., Huang Y., Zhou J., Xu G., Wang T. (2017). Differential Regulation of Two-Tiered Plant Immunity and Sexual Reproduction by ANXUR Receptor-Like Kinases. Plant Cell.

[213] Baumberger N., Doesseger B., Guyot R., Diet A., Parsons R.L., Clark M.A., Simmons M.P., Bedinger P., Goff S.A., Ringli C. (2003). Whole-genome comparison of leucine-rich repeat extensins in Arabidopsis and rice. A conserved family of cell wall proteins form a vegetative and a reproductive clade. Plant Physiol..

[214] Lamport D.T.A., Kieliszewski M.J., Chen Y., Cannon M.C. (2011). Role of the extensin superfamily in primary cell wall architecture. Plant Physiol..

[215] Qi X., Behrens B.X., West P.R., Mort A.J. (1995). Solubilization and partial characterization of extensin fragments from cell walls of cotton suspension cultures. Evidence for a covalent cross-link between extensin and pectin. Plant Physiol..

[216] Ringli C. (2010). The hydroxyproline-rich glycoprotein domain of the Arabidopsis LRX1 requires Tyr for function but not for insolubilization in the cell wall. Plant J..

[217] Wolf S., Mravec J., Greiner S., Mouille G., Höfte H. (2012). Plant Cell Wall Homeostasis Is Mediated by Brassinosteroid Feedback Signaling. Curr. Biol..

[218] Moussu S., Broyart C., Santos-Fernandez G., Augustin S., Wehrle S., Grossniklaus U., Santiago J. (2020). Structural basis for recognition of RALF peptides by LRX proteins during pollen tube growth. Proc. Natl. Acad. Sci. USA.

[219] Nakhamchik A., Zhao Z., Provart N.J., Shiu S.-H., Keatley S.K., Cameron R.K., Goring D.R. (2004). A comprehensive expression analysis of the Arabidopsis proline-rich extensin-like receptor kinase gene family using bioinformatic and experimental approaches. Plant Cell Physiol..

[220] Cho H.-T.T. (2016). Arabinogalactan protein motif-containing receptor-like kinases are likely to play the negative feedback factor to maintain proper root hair length. Plant Signal. Behav..

[221] Hwang Y., Lee H., Lee Y.-S.S., Cho H.-T.T. (2016). Cell wall-associated ROOT HAIR SPECIFIC 10, a proline-rich receptor-like kinase, is a negative modulator of Arabidopsis root hair growth. J. Exp. Bot..

[222] Tian J., Han L., Feng Z., Wang G., Liu W., Ma Y., Yu Y., Kong Z. (2015). Orchestration of microtubules and the actin cytoskeleton in trichome cell shape determination by a plant-unique kinesin. eLife.

[223] Basu D., Tian L., DeBrosse T., Poirier E., Emch K., Herock H., Travers A., Showalter A.M. (2016). Glycosylation of a Fasciclin-Like Arabinogalactan-Protein (SOS5) mediates root growth and seed mucilage adherence via a cell wall receptor-like kinase (FEI1/FEI2) Pathway in Arabidopsis. PLoS ONE.

[224] Seifert G.J. (2021). The FLA4-FEI pathway: A unique and mysterious signaling module related to cell wall structure and stress signaling. Genes.

[225] Arioli T., Peng L., Betzner A.S., Burn J., Wittke W., Herth W., Camilleri C., Höfte H., Plazinski J., Birch R. (1998). Molecular analysis of cellulose biosynthesis in Arabidopsis. Science.

[226] Schindelman G., Morikami A., Jung J., Baskin T.I., Carpita N.C., Derbyshire P., McCann M.C., Benfey P.N. (2001). COBRA encodes a putative GPI-anchored protein, which is polarly localized and necessary for oriented cell expansion in Arabidopsis. Genes Dev..

[227] Harpaz-Saad S., McFarlane H.E., Xu S., Divi U.K., Forward B., Western T.L., Kieber J.J. (2011). Cellulose synthesis via the FEI2 RLK/SOS5 pathway and CELLULOSE SYNTHASE 5 is required for the structure of seed coat mucilage in Arabidopsis. Plant J..

[228] Steinwand B.J., Xu S., Polko J.K., Doctor S.M., Westafer M., Kieber J.J. (2014). Alterations in auxin homeostasis suppress defects in cell wall function. PLoS ONE.

[229] Chevalier D., Batoux M., Fulton L., Pfister K., Yadav R.K., Schellenberg M., Schneitz K. (2005). STRUBBELIG defines a receptor kinase-mediated signaling pathway regulating organ development in Arabidopsis. Proc. Natl. Acad. Sci. USA.

[230] Lin L., Zhong S.-H., Cui X.-F., Li J., He Z.-H. (2012). Characterization of temperature-sensitive mutants reveals a role for receptor-like kinase SCRAMBLED/STRUBBELIG in coordinating cell proliferation and differentiation during Arabidopsis leaf development. Plant J..

[231] Kwak S.-H., Schiefelbein J. (2007). The role of the SCRAMBLED receptor-like kinase in patterning the Arabidopsis root epidermis. Dev. Biol..

[232] Kwak S.-H., Woo S., Lee M.M., Schiefelbein J. (2014). Distinct signaling mechanisms in multiple developmental pathways by the SCRAMBLED receptor of Arabidopsis. Plant Physiol..

[233] Beauzamy L., Derr J., Boudaoud A. (2015). Quantifying hydrostatic pressure in plant cells by using indentation with an atomic force microscope. Biophys. J..

